# A combination of NLR and sST2 is associated with adverse cardiovascular events in patients with myocardial injury induced by moderate to severe acute carbon monoxide poisoning

**DOI:** 10.1002/clc.23550

**Published:** 2021-01-26

**Authors:** Qian Liu, Xun Gao, Qingmian Xiao, Baoyue Zhu, Yongjian Liu, Yongyan Han, Weizhan Wang

**Affiliations:** ^1^ Department of EICU Harrison International Peace Hospital Affiliated to Hebei Medical University Hengshui China; ^2^ Department of Emergency General Ward Harrison International Peace Hospital Affiliated to Hebei Medical University Hengshui China; ^3^ Department of Emergency Medicine Harrison International Peace Hospital Affiliated to Hebei Medical University Hengshui China

**Keywords:** acute carbon monoxide poisoning, adverse cardiovascular events, myocardial injury, NLR, sST2

## Abstract

**Background:**

Indicators of adverse cardiovascular events in patients with acute carbon monoxide (CO) poisoning‐induced myocardial injury have not yet been elucidated.

**Hypothesis:**

This study aimed at determining the risk factors for adverse cardiovascular events in patients with acute CO poisoning‐induced myocardial injury.

**Methods:**

We enrolled patients with moderate‐to‐severe acute CO poisoning‐induced myocardial injury. Based on the occurrence of adverse cardiovascular events, the patients were assigned into event and non‐event group. Binary logistic regression analysis was performed to analyze the potential risk factors for cardiovascular adverse events.

**Results:**

A total of 413 eligible patients were enrolled. Among them, 61 (14.8%) patients presented adverse cardiovascular events and were assigned to the event group while 352 patients were assigned to the non‐event group. Univariate analysis revealed that cTnI, Lac, and NLR levels at admission and sST2 at day 3 in the event group were significantly higher compared to those in the non‐event group. Subsequent multivariate analysis revealed that sST2 at day 3 and NLR at admission were independent risk factors for adverse cardiovascular events in patients with acute CO poisoning‐induced myocardial injury. Finally, the sensitivity, specificity, and AUC of sST2 at day 3 combined with NLR for event prediction were 79.5%, 82.8%, and 0.858, respectively.

**Conclusion:**

A combination of sST2 at day 3 and NLR is a potential predictor for the occurrence of adverse cardiovascular events in patients with acute CO poisoning‐induced myocardial injury. Therefore, cardiovascular risk stratification should be taken into consideration, especially in patients with acute CO poisoning‐induced myocardial injury.

## 1 INTRODUCTION

Acute carbon monoxide (CO) poisoning‐induced myocardial injury is often neglected due to its hidden and reversible process.^1^ Acute CO poisoning can lead to adverse cardiovascular events, such as, serious arrhythmia, acute heart failure, myocardial infarction, and even sudden death.^2^, ^3^

Several different mechanisms by which acute CO poisoning may induce myocardial injury have been established. Firstly, the reduction in oxygen delivery caused by acute CO poisoning can be compensated by an increase in cardiac output and oxygen uptake; however, adverse cardiovascular events might occur when this is decompensated. Secondly, this poisoning can lead to a systemic inflammatory reaction, resulting in extensive endothelial dysfunction and, ultimately, cardiac function inhibition.^4^ Thirdly, mitochondrial oxidative phosphorylation can be destroyed by acute CO poisoning, which leads to myocardial stunning, resulting in cardiac function injury, arrhythmia, angina pectoris, and eventually acute myocardial infarction.^5^, ^6^ Fourthly, this poisoning can lead to sympathetic nerve excitation that stimulates catecholamine production. The microvascular spasm or dysfunction induced by catecholamines can lead to either myocardial stunning or direct toxicity to myocardial cells.^4^

Studies have documented that there are two peaks in acute CO poisoning‐induced cardiovascular system injury. The first peak appears immediately after poisoning, which indicates the direct and indirect toxic effects of CO on the myocardium while the second peak emerges on day 3 after poisoning, and is associated with reperfusion injury.^7^, ^8^ It has also been reported that both early poisoning/hypoxia and subsequent ''ischemia–reperfusion'' process can activate inflammatory cells, leading to inflammatory injury to the cardiovascular system.^9^

However, to date, very few studies have evaluated the predictive indicators of adverse cardiovascular events in patients with acute CO poisoning‐induced myocardial injury. There is an urgent medical need to elucidate on the indicators that can predict adverse cardiovascular events in such patients. In particular, the neutrophil to lymphocyte ratio (NLR) is a new inflammatory‐associated biomarker that has been correlated with the severity and prognosis of many cardiovascular diseases. Neutrophils represent endothelial injury and platelet aggregation during the inflammatory stage, while reduced lymphocytes reflect physiological stress and poor health status. Therefore, NLR is a balance between inflammation and stress response pathways.^10^, ^11^ Furthermore, it is an inexpensive, easy to obtain, and widely available marker. Conversely, ST2 is a member of the interleukin (IL)‐1 receptor superfamily. Specifically, soluble ST2 (sST2) is a valuable biomarker in acute cardiovascular disease. Multiple studies have demonstrated its prognostic value in the clinical outcomes of cardiovascular disease patients.^12^ Therefore, this study aimed at determining the risk factors for adverse cardiovascular events in patients with acute CO poisoning‐induced myocardial injury, so as to inform on the early monitoring and intervention of adverse cardiovascular events in such patients.

## 2 MATERIALS AND METHODS

### 2.1 Study participants

We retrospectively reviewed the medical records of patients with moderate to severe acute CO poisoning‐associated myocardial injury, who had been admitted at the Department of Emergency or Emergency Intensive Care Unit (EICU) of Harrison International Peace Hospital between November, 2015 and February, 2020. Moderate to severe acute CO poisoning was diagnosed by the criteria of occupational acute carbon monoxide poisoning GBZ23‐2002,^13^ while myocardial injury was diagnosed in conformity with the diagnostic criteria for occupational acute toxic cardiopathy caused by chemicals GBZ 74–2009.^14^ Patients were eligible for this study if they had a history of CO exposure, with coma symptoms, positive cardiac troponin I (cTnI) after 3 hours of admission, and admitted to the hospital within 24 hours of acute CO poisoning onset.

The exclusion criteria were: (a). Patients younger than 18 years, with acute pulmonary edema, cardiogenic shock, atrial fibrillation, paroxysmal supraventricular tachycardia, ventricular tachycardia, atrioventricular block, ventricular fibrillation, or other cardiovascular events; (b) Patients with autoimmune diseases, such as, rheumatoid arthritis, systemic lupus erythematosus, ankylosing spondylitis; (c) Those who had been administered with immunosuppressive drug within 3 months before enrollment; (d) Patients with coronary heart diseases, cardiomyopathy, or chronic cardiac insufficiency; (e) Patients with bronchial asthma, chronic obstructive pulmonary disease, or pulmonary interstitial fibrosis; (f) Those with hematological diseases; (g) Those with serious infections at admission; and (h). Those with a malignant tumor.

### 2.2 Procedures

All patients were subjected to electrocardiographic monitoring (24‐h rhythm recording), oxygen inhalation, and hyperbaric oxygen treatment. Those who could not receive hyperbaric oxygen therapy were given intermittent ventilator assisted pure oxygen inhalation in order to prevent and treat brain edema and myocardial nutrition.

Subsequently, the patients were assigned into two categories, event and non‐event groups, based on whether adverse cardiovascular events occurred or not within 1 month after hospital admission. The cardiovascular events included serious arrhythmia, heart failure, angina pectoris, and myocardial infarction, cardiogenic shock, cardiac arrest, as well as all‐cause death. Serious arrhythmia was defined as newly emerging symptoms after admission, such as, atrial fibrillation, paroxysmal supraventricular tachycardia, ventricular tachycardia, ventricular fibrillation, and atrioventricular block recorded using electrocardiography. Notably, if two or more events occurred at the same time or in succession, the events would only be recorded once.

### 2.3 Assessment

Venous blood samples were obtained at admission. We subsequently performed blood routine examinations to detect neutrophil and lymphocyte counts. Then, we calculated the NLR values. Moreover, 2 mL elbow venous blood was drawn at admission and 3 days after admission, placed in the red cap catheter and sent to the central laboratory. The levels of sST2 were measured using the enzyme‐linked immunosorbent assay (ELISA) kit (Ruidi Biotech, Shanghai, China). Samples collected at night and which could not be sent for examination in time were stored at 4°C in the refrigerator.

Finally, the baseline information of patients at admission, including gender, age, duration of coma, history of hypertension and diabetes, whether subjected to tracheal intubation or not, acute physiology and chronic health inquiry (APACHE II) score, lactate (LAC) levels, cTnI, creatine kinase isoenzyme (CK‐MB), creatine kinase (CK), as well as N‐terminal pro‐brain natriuretic peptide (NT‐proBNP) was collected.

### 2.4 Statistical analysis

All statistical analyses were performed using the SPSS software, version 21.0. A nonparametric K‐S test was used to assess data normality. The variable data are expressed as mean ± standard deviation (SD). Comparison between groups was performed using a *t* test, while the attributes data were analyzed using the *χ* ^2^ test. The correlations between risk factors and cardiovascular events were determined using binary logistic regression and the receiver operating characteristic (ROC) curve. p≤0.05 was considered statistically significant.

## 3 RESULTS

### 3.1 Baseline characteristics

In this study, we enrolled a total of 413 eligible patients with acute CO poisoning and myocardial injury. Among them, 61 (14.8%) patients exhibited cardiovascular events, including 26 cases of atrial fibrillation requiring drug intervention, 5 ventricular tachycardia cases, 17 heart failure cases, 6 sudden death cases, 3 deaths due to multiple organ failure, and 4 cases of chest pain combined with myocardial infarction‐like electrocardiography changes. These 61 patients were assigned to the event group, while the remaining 352 patients were assigned to the non‐event group (Table 1).

**TABLE 1:** Baseline characteristics

	Non‐event group (n = 352)	Event group (n = 61)	*χ* ^2^/T test	p value
Age (years)	60.26 ± 10.32	61.32 ± 11.24	0.316	0.723
Male	197 (56.0)	39 (63.9)	0.892	0.315
Duration of coma (hours)	9.06 ± 7.89	12.66 ± 8.53	2.232	0.032
APACHE II score	12.85 ± 4.17	15.72 ± 5.16	2.552	0.011
Endotracheal intubation	33 (9.4)	5 (8.2)	0.002	0.649
Hypertension	148 (42.0)	33 (54.1)	3.217	0.086
Diabetes	73 (20.7)	18 (29.5)	0.931	0.228

*Note:* Data are mean ± SD or n (%). APACHE II, Acute Physiology, and Chronic Health Enquiry II.

Moreover, coma duration in the event group was significantly longer than that of the non‐event group (p = 0.032). Likewise, the APACHE II score of the event group was significantly higher compared with that of the non‐event group (p = 0.011). There was no significant difference between the two groups in terms of gender, age, incidence of endotracheal intubation, number of patients with hypertension, and number of patients with diabetes.

### 3.2 Univariate analysis of cardiovascular indicators associated with acute CO poisoning ‐induced adverse cardiovascular events

We assessed the levels of several cardiovascular indicators to establish the correlations that might exist between adverse cardiovascular events and changes in these parameters. Notably, the levels of; cTnI (event vs non‐event group, 0.77 ± 0.41 vs 0.43 ± 0.16 ng/mL, p = 0.013), Lac (3.02 ± 2.44 vs 2.32 ± 1.96 mmoL/L, p = 0.048), NLR at admission (13.92 ± 4.51 vs 9.11 ± 5.72, p<0.001), and sST2 at day 3 (64.76 ± 19.54 vs 38.91 ± 10.25 ng/mL, p<0.001) in the event group were significantly higher than those in the non‐event group (Table 2). However, there were no significant differences between the two groups in; sST2 at admission (event vs non‐event group, 250.81 ± 76.82 vs 221.43 ± 90.10 ng/mL, p = 0.357), CK (3996.17 ± 602.13 vs 2963.32 ± 475.25 U/L, p = 0.202), CK‐MB (98.24 ± 16.62 vs 79.58 ± 11.26 U/L, p = 0.128), and NT‐proBNP (2206.11 ± 325.23 vs 1832.53 ± 335.41 pg/mL, p = 0.446).

**TABLE 2:** Comparisons of cardiovascular parameters between event group and non‐event group

	Non‐event group (n = 352)	Event group (n = 61)	T test	p value
cTnI (ng/ml)	0.43 ± 0.16	0.77 ± 0.41	3.232	0.013
CK (U/l)	2963.32 ± 475.25	3996.17 ± 602.13	1.825	0.202
CK‐MB (U/l)	79.58 ± 11.26	98.24 ± 16.62	2.102	0.128
NT‐proBNP (pg/ml)	1832.53 ± 335.41	2206.11 ± 325.23	0.921	0.446
Lac (mmol/l)	2.32 ± 1.96	3.02 ± 2.44	2.011	0.048
NLR	9.11 ± 5.72	13.92 ± 4.51	4.466	<0.001
sST2 at admission (ng/ml)	221.43 ± 90.10	250.81 ± 76.82	0.814	0.357
sST2 at day 3 (ng/ml)	38.91 ± 10.25	64.76 ± 19.54	7.260	<0.001

*Note:* Data are mean ± SD.

### 3.3 Multivariate analysis of cardiovascular indicators associated with acute CO poisoning‐induced adverse cardiovascular events

All variables with p<0.2 in the univariate analysis were included in the subsequent binary logistic regression multivariate analysis. Our findings showed that sST2 at day 3 (OR = 1.084, 95% CI 1.049–1.116, p<0.001) and NLR at admission (OR = 1.277, 95% CI 1.120–1.439, p<0.001) were independent risk factors for adverse cardiovascular events in patients with myocardial injury caused by acute CO poisoning (Table 3).

**TABLE 3:** Multivariable analysis of ACOP‐induced myocardial injury‐associated cardiovascular parameters

	β value	Standard error of Mean	Wald	p value	OR	95% CI
sST2 at day 3	0.080	0.017	29.132	<0.001	1.084	1.049–1.116
NLR	0.241	0.065	14.952	<0.001	1.277	1.120–1.439
APACHE II score	0.120	0.053	4.819	0.027	1.128	1.011–1.265

**FIGURE 1:**
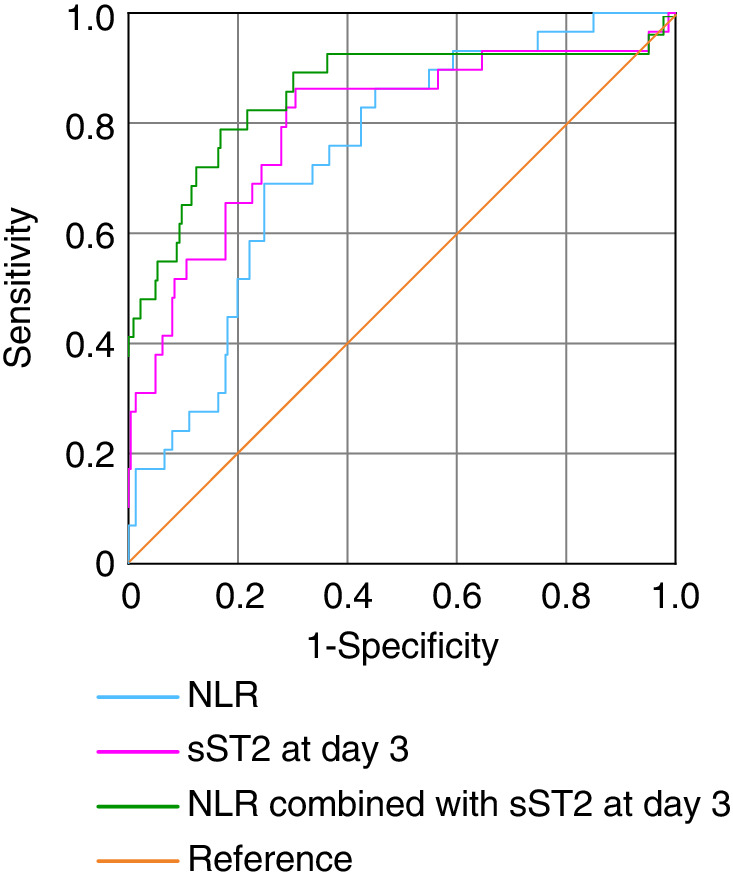
The receiver operating characteristic for the prediction of cardiovascular events using neutrophil to lymphocyte ratio combined with sST2 at day 3 in patients with acute CO poisoning‐induced myocardial injury

We then evaluated the values of sST2 on day 3 and NLR at admission in the prediction of adverse cardiovascular events in patients with acute CO poisoning‐induced myocardial injury (Figure 1, Table 4). The ROC curve revealed that the best NLR and sST2 (at day 3) cutoff values were 12.05 and 44.21 ng/mL, respectively. Using the 12.05 NLR cutoff point, the sensitivity and specificity in predicting cardiovascular events were 69.2% and 75.0%, respectively (AUC_NLR_ = 0.739). Additionally, using the 44.21 ng/mL sST2 (at day 3) cutoff point, the sensitivity and specificity in predicting adverse cardiovascular events were 82.9% and 70.9%, respectively (AUC_sST2 at day 3_ = 0.789). Finally, the sensitivity and specificity of sST2 (at day 3) combined with NLR in predicting cardiovascular adverse events were 79.5% and 82.8%, respectively (AUC = 0.858).

**TABLE 4:** Prediction of cardiovascular events using NLR combined with sST2 at day 3 in patients with ACOP‐induced myocardial injury

	AUC	Youden index	95% CI	p value	Best cutoff	Sensitivity	Specificity
NLR	0.739	0.439	0.648–0.827	<0.001	12.05	69.2%	75.0%
sST2 at day 3	0.789	0.537	0.685–0.891	<0.001	44.21	82.9%	70.9%
NLR combined with sST2 at day 3	0.858	0.621	0.762–0.951	<0.001	/	79.5%	82.8%

Abbreviation: AUC, area under curve.

## 4 DISCUSSION

In this study, we found that sST2 at day 3 and NLR at admission were independent risk factors for adverse cardiovascular events in patients with acute CO poisoning‐induced myocardial injury. We also found that a combination of sST2 at day 3 and NLR is a potential predictor for the occurrence of adverse cardiovascular events in patients with acute CO poisoning‐induced myocardial injury. Overall, this study informs on treatment and reduction of the risk of adverse cardiovascular events in patients with acute CO poisoning‐induced myocardial injury.

Cardiac troponin I (cTnI) is one of the specific biomarkers for myocardial injury. When myocardial cells are injured, cTnI is rapidly released from the cytoplasm and into the blood. Its serum concentration levels reflects the myocardial injury grade.^15^ Consistent with previous studies, we found that cTnI levels in the event group were significantly higher compared with the non‐event group (p = 0.013). However, based on the results of binary logistic regression analysis, we did not include cTnI in the prediction model for adverse cardiovascular events. This is because, although cTnI is a reliable indicator of myocardial injury, it was not an independent predictor of adverse cardiovascular events in patients with acute CO poisoning‐induced myocardial injury. Furthermore, this may be attributed to the fact that most patients with moderate to severe acute CO poisoning are found in an unconscious state by others, and hence the exact onset time of poisoning remains elusive. Therefore, patients may be in various stages of troponin elevation, (such as, just beginning to rise, already at the peak, or after the peak) at the time of examination, making it difficult to detect the peak concentration of cTnI in clinical practice.

NLR possesses benefits such as easy acquisition, low cost, and strong generalization. An elevated NLR level is a risk factor for adverse cardiovascular events in patients with acute coronary syndrome.^16^ In this study, NLR in the event group was significantly higher than that in the non‐event group (*p*<0.001). A plausible explanation is that inflammation was involved in the mechanism of acute CO poisoning‐induced myocardial injury. In addition, elevated neutrophil counts reflect the deterioration of systemic inflammation, while low lymphocyte counts imply a decline in immune regulation ability and an increase in stress. Therefore, elevated NLR reflects inflammatory imbalance that induces adverse cardiovascular events in these patients.

According to literature, a single pathophysiological pathway cannot fully explain the underlying mechanism of myocardial injury. Of note, ST2 plays an important immune regulatory role in a variety of inflammatory processes, and it can be expressed in myocardial cells during ischemia, hypoxia, or mechanical stress. As a potential biomarker that can reflect myocardial stretch, fibrosis, and body inflammation, sST2 can predict the occurrence of adverse cardiovascular events in patients with myocardial infarction, acute coronary syndrome, heart failure, and sepsis.^17^, ^18^, ^19^ We found that at admission, the ST2 levels between the event and non‐event groups were similar. However, at day 3 after admission, there was a significant difference in ST2 levels between the two groups (p<0.001). Remarkably, binary logistic regression analysis revealed that sST2 level at day 3 was an independent risk factor for predicting adverse cardiovascular events. This finding is in tandem with that of the PRAISE‐2 study that involved patients with chronic heart failure and cardiac functions of grade 3/4. In the PRAISE‐2 study, changes in sST2 levels rather than the baseline sST2 level were found to be a predictive factor of death within 90 days after heart failure.^20^ Regarding the release pattern of sST2, Eggers et al. reported that sST2 serum levels increased after 2 to 4 h of the onset of non–ST‐segment elevation myocardial infarction (NSTEMI), reached the peak after 6 to 17 h, and then gradually decreased to a steady‐state.^21^ However, the release pattern of sST2 in rheumatic immune diseases, pulmonary interstitial fibrosis, and other diseases has not been reported. In our pilot study, we found that sST2 levels in patients with acute CO poisoning‐induced myocardial injury increased significantly at admission, but rapidly decreased 3 days later. Therefore, in this study, the time of admission and day 3 were selected as the time points of serum sST2 detection for adverse cardiovascular events prediction. We found that on day 3, the sensitivity and specificity of sST2 in predicting adverse cardiovascular events were 82.9% and 70.9%, respectively.

However, despite these promising results, our study is associated with several shortcomings. Firstly, this was a single‐center study with a relatively small sample size. Secondly, this is a retrospective study conducted by reviewing the medical records of all enrolled patients, which may not be perfect. Thirdly, we only detected sST2 levels at admission and at 3 days after admission. Studies using more time points are needed to elucidate the predictive value of sST2 for adverse cardiovascular events.

In conclusion, an adverse cardiovascular event is a common consequence of moderate to severe acute CO poisoning‐induced myocardial injury. This study aimed at evaluating the early‐warning indicators associated with myocardial injury, which may guide doctors to administer early and adequate treatment in high‐risk patients, to improve ischemia and hypoxia as early as possible, and to avoid or reduce the occurrence of myocardial injury. Based on the findings from this study, we recommend that patients exposed to CO should be examined for myocardial injury. Furthermore, we suggest that cardiovascular risk stratification should be taken into consideration, particularly in patients diagnosed with acute CO poisoning‐induced myocardial injury.

## CONFLICT OF INTEREST

All authors completed the ICMJE uniform disclosure form. We declare no competing interests.

## AUTHOR CONTRIBUTION

Conception and design: Qian Liu, Weizhan Wang. Administrative support: Qian Liu, Weizhan Wang. Provision of study materials or patients: Xun Gao, Qingmian Xiao, Baoyue Zhu, Yongjian Liu, Yongyan Han. Data collection and assembly: Xun Gao, Qingmian Xiao, Baoyue Zhu, Yongjian Liu, Yongyan Han. Data analysis and interpretation: Qian Liu, Xun Gao, Qingmian Xiao, Baoyue Zhu, Yongjian Liu, Yongyan Han, Weizhan Wang. Manuscript writing: All authors. Final manuscript approval: All authors.

## ETHICAL STATEMENT

The authors are accountable for all aspects of this study in ensuring that questions related to the accuracy or integrity of any part of this study are appropriately investigated and resolved. This study was performed in conformity with the Declaration of Helsinki and was approved by the ethics committee of Harrison International Peace Hospital. All study participants gave their informed written consent.
